# Copper-catalyzed functionalization of enynes

**DOI:** 10.1039/d0sc04012f

**Published:** 2020-10-07

**Authors:** Quentin Dherbassy, Srimanta Manna, Fabien J. T. Talbot, Watcharapon Prasitwatcharakorn, Gregory J. P. Perry, David J. Procter

**Affiliations:** Department of Chemistry, University of Manchester Oxford Road Manchester M13 9PL UK david.j.procter@manchester.ac.uk www.twitter.com/GroupProcter https://www.proctergroupresearch.com/

## Abstract

The copper-catalyzed functionalization of enyne derivatives has recently emerged as a powerful approach in contemporary synthesis. Enynes are versatile and readily accessible substrates that can undergo a variety of reactions to yield densely functionalized, enantioenriched products. In this perspective, we review copper-catalyzed transformations of enynes, such as boro- and hydrofunctionalizations, copper-mediated radical difunctionalizations, and cyclizations. Particular attention is given to the regiodivergent functionalization of 1,3-enynes, and the current mechanistic understanding of such processes.

## Abstract 2

The copper-catalyzed functionalization of enynes is a powerful approach to yield densely functionalized products. This review covers various transformations, such as boro- and hydrofunctionalizations, copper-mediated radical difunctionalizations, and cyclizations.

## Graphical abstract

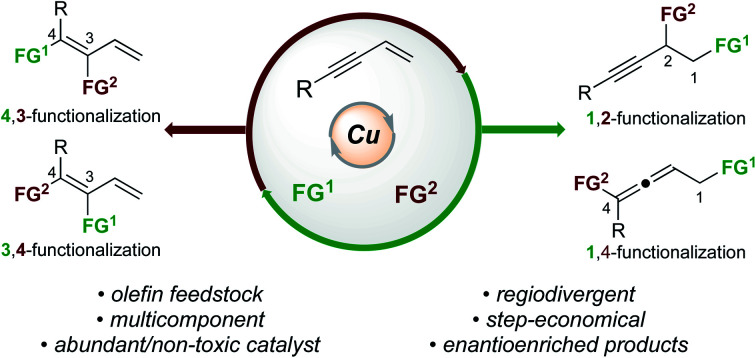

## 1. Introduction

The desire to build complex, functionalized molecules rapidly and efficiently from simple precursors is a recurring theme in modern organic chemistry. Many envision a sustainable future in which the processing of feedstock substrates in catalytic and step-/atom-economical transformations will allow new regions of chemical space to be explored.^1^ The transition-metal catalyzed functionalization of olefins is seen as an approach with the potential to contribute to such a future,^2^ as it has proved capable of delivering high-value, multifunctionalized, stereodefined products. Copper catalysts hold particular promise for fulfilling goals pertaining to sustainability, as copper is more abundant^3^ and less toxic^4^ than other transition metals. Indeed, various olefins^5^ have been transformed into densely functionalized products^6^ using copper catalysis. In particular, enynes are highly versatile substrates. They can be obtained using numerous, efficient catalytic methods^7^ and their ambident reactivity has been exploited to deliver a multitude of products.^5*f*,8^

**Scheme 1:**
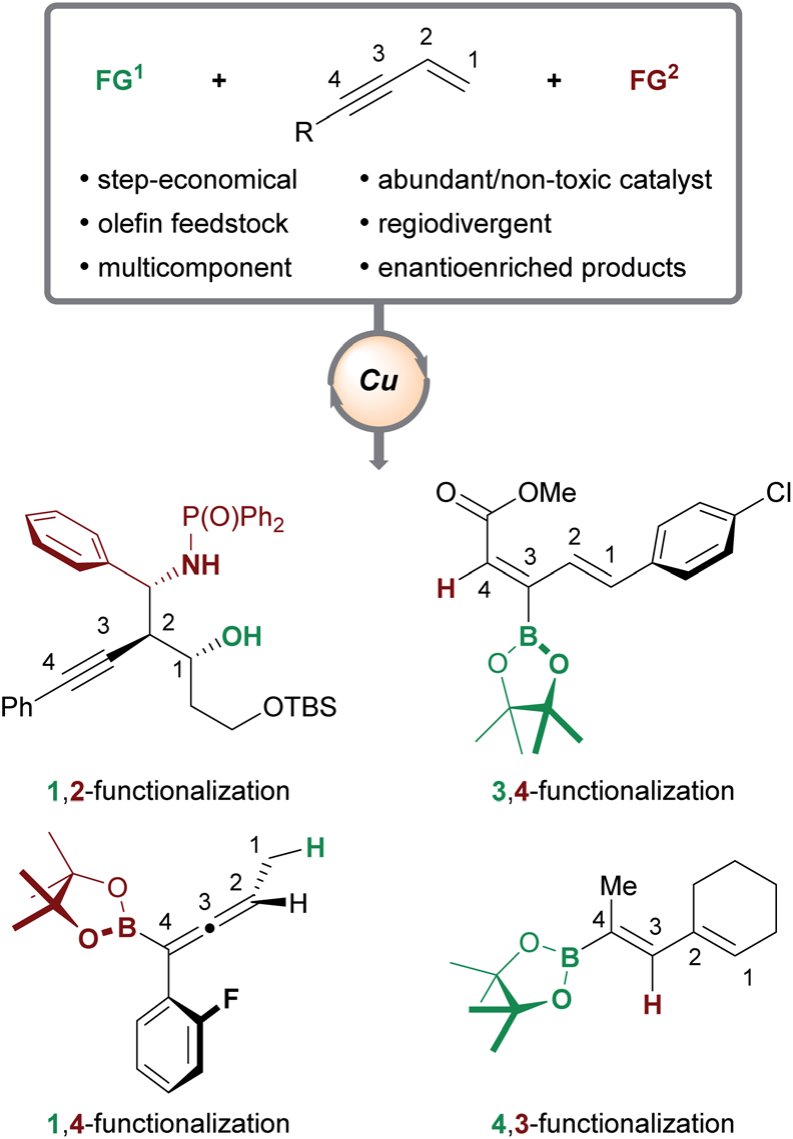
Copper-catalyzed functionalization of enynes.

Herein, we will review the copper-catalyzed functionalization of enynes. This fast-growing field has seen the implementation of various strategies in copper catalysis, such as hydro- and borofunctionalizations, multicomponent reactions, radical difunctionalizations, and cyclizations. The copper-catalyzed functionalization of enynes will be discussed in four sections: (1) borofunctionalization, where a copper-boryl complex is the catalytically active species; (2) hydrofunctionalization, where a copper-hydride complex is the catalytically active species; (3) copper-mediated radical difunctionalization, and; (4) copper-catalyzed functionalization of non-conjugated 1,*n*-enynes. Generally, 1,3-enynes are the most common substrates in these processes and a variety of highly useful products are accessible (Scheme 1); for example, enantioenriched, (homo)propargylic compounds (1,2-functionalization), allenes (1,4-functionalization), and dienes (4,3-/3,4-functionalization), are important in synthetic^9^ and/or medicinal chemistry.^10^

## 2. Mechanistic principles

**Scheme 2:**
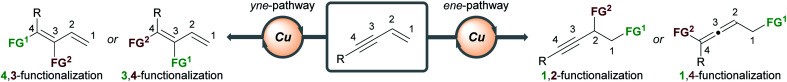
Copper-catalyzed regiodivergent functionalization of enynes.

The outcome of the copper-catalyzed functionalization of 1,3-enynes is determined by the interplay of several factors at different stages of the catalytic cycle: the chemo- (alkene *vs.* alkyne) and regioselectivity (*e.g.* 1,2- *vs.* 2,1-addition) of the first functional group (FG^1^) addition, the possible isomerization of organocopper intermediates, and the mechanism of the second functional group (FG^2^) addition. Details of these mechanisms will be presented when appropriate and when experimental evidence or DFT calculations are available. In general terms, the possible products can be grouped into two families (Scheme 2) – it is the chemoselectivity of the reaction with the first functional group (FG^1^) that determines which family of product forms. Other products are theoretically possible, however, Scheme 2 summarises the types of products that have been accessed to date. If the initial functionalization occurs at the olefinic component of the enyne (the *ene*-pathway), the (homo)propargylic/allenic family of products is formed. Alternatively, reaction of FG^1^ with the alkyne component (*yne*-pathway) leads to the diene family of products. Within each family are sub-groups that are accessed through different modes of addition to the enyne. For example, in the *ene*-pathway, 1,2-functionalization leads to (homo)propargylic products, whereas 1,4-functionalization leads to allenic products. The notation used to describe the type of functionalization refers to the position of the attached functional groups in the product, *i.e.* the (homo)propargylic product is said to form *via* a 1,2-functionalization as the first functional group (FG^1^) is attached to C1 and the second functional group (FG^2^) is attached to C2. Using the same concepts, the *yne*-pathway leads to diene products of either 4,3-or 3,4-functionalization. This is a simplified model for describing the outcomes of these reactions and a similar nomenclature will be used throughout this perspective. There are numerous pathways by which enynes can react and a detailed description of each mechanistic pathway is beyond the scope of this review, however, key aspects will be highlighted.

## 3. Borofunctionalization

Boron-containing compounds are involved in 11% of the C–C bond forming reactions used in process chemistry.^1*d*^ Since the seminal reports of Miyaura^11^ and Hosomi^12^ on the borylation of enones, the copper-catalyzed borylation of olefins has become a versatile starting point for the design of important multicomponent reactions.^5*c*,5*d*,13^ Thus, it is not surprising to find these processes at the heart of several seminal reports on the copper-catalyzed functionalization of 1,3-enynes.

### 3.1. Regiodivergent processes

**Scheme 3:**
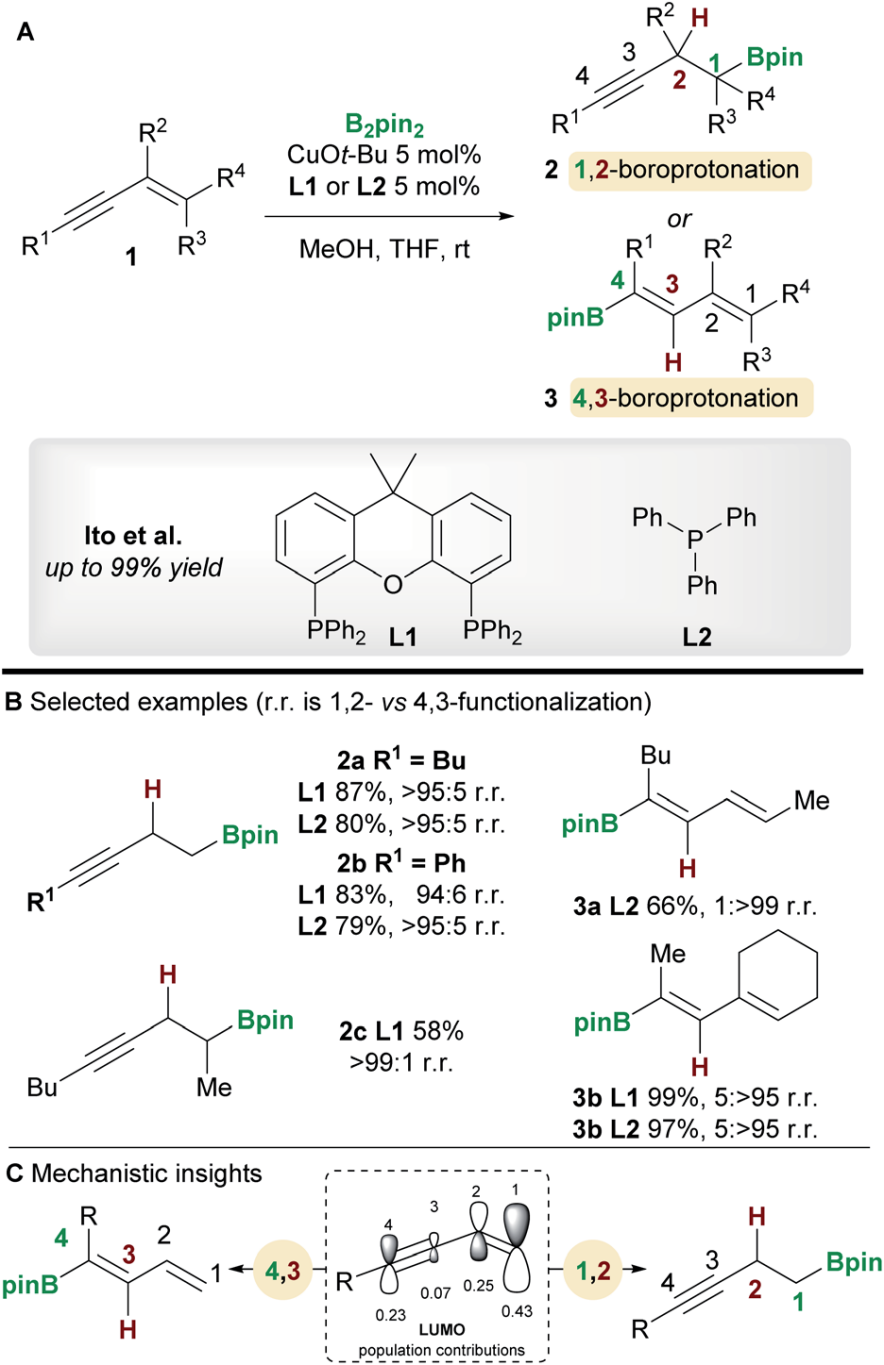
Ito's regiodivergent boroprotonation of 1,3-enynes.

The copper-catalyzed boroprotonation of 1,3-enynes **1** reported by Ito and co-workers^14^ in 2011 introduced some useful concepts concerning the regioselectivity of enyne functionalization (Scheme 3A). The system used a Cu-Bpin catalyst, formed *in situ* from a Cu-alkoxide and B_2_pin_2_, and MeOH as a proton source. The reaction of relatively simple enynes, for example butyl-1,3-enyne **1a** and phenyl-1,3-enyne **1b**, provided the 1,2 products **2a**/**2b** with high regioselectivity with ligands **L1** and **L2** (Scheme 3B). The authors then explored the effect of substitution on the alkene moiety of the enyne and observed a ligand-controlled regioselectivity switch. Thus, 1-substituted enynes gave the 1,2-product **2c** with the bidentate XantPhos ligand **L1**, whereas the 4,3-product **3a** was obtained when using monodentate PPh_3_**L2**. Conversely, 1,2-disubstituted enynes always gave the 4,3-product **3b** no matter which ligand was used, possibly due to the steric hindrance around the olefin. The authors also reported a moderately enantioselective 1,2-boroprotonation. Frontier orbital population analysis from DFT calculations was used to explain the regioselectvity of these results (Scheme 3C). The most important interaction to consider is between the HOMO of the copper-boryl complex and the LUMO of the enyne. In this regard, the larger coefficients at C1 and C4 explain the preference for 1,2-/4,3-functionalization. However, a detailed discussion of the observed regiodivergency was not provided.

**Scheme 4:**
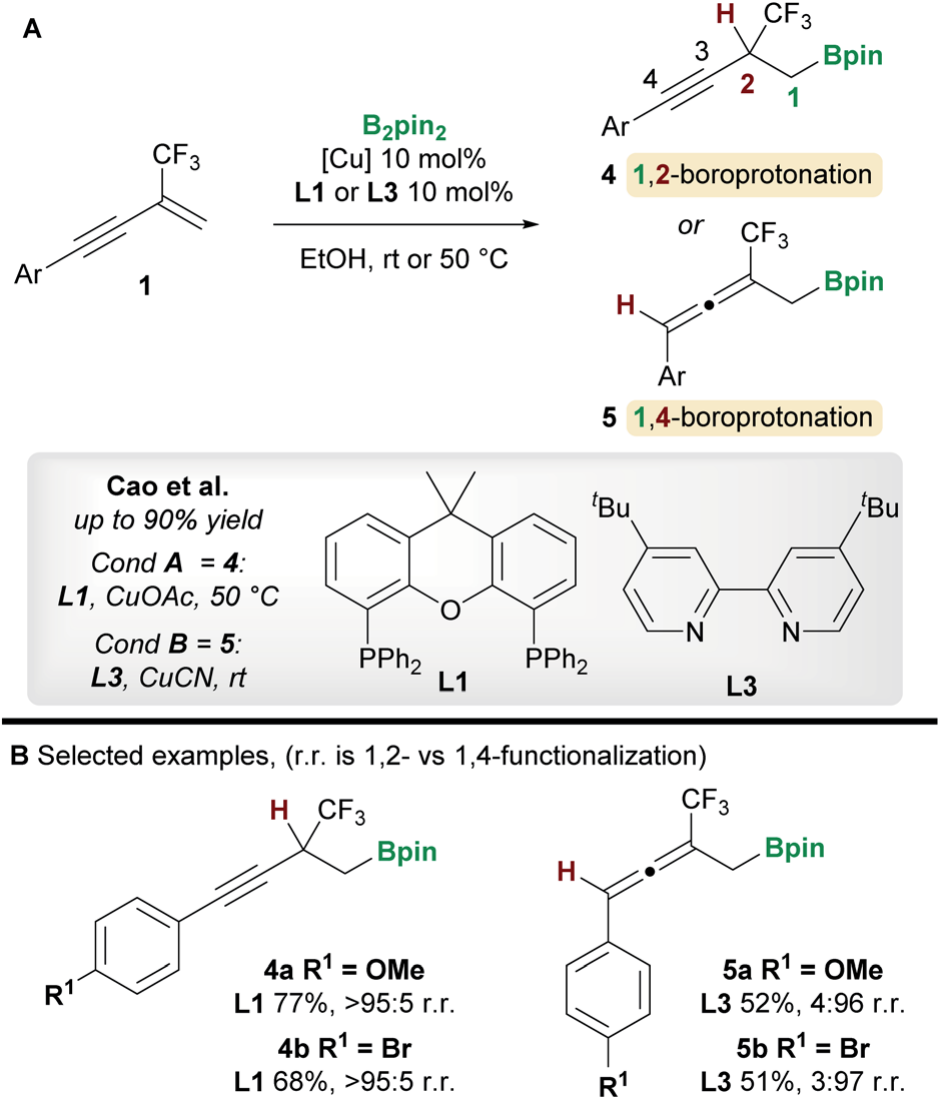
Cao's regiodivergent boroprotonation of trifluoromethyl-substituted 1,3-enynes.

In 2020, a regiodivergent boroprotonation of 2-trifluoromethyl-1,3-enynes **1** was reported by Cao and co-workers (Scheme 4).^15^ In accordance with the previous work of Ito *et al.* (Scheme 3), they found that XantPhos **L1** promoted the 1,2-boroprotonation of 2-substituted 1,3-enynes in good yields and with complete regiocontrol (see **4***vs.***2**). Conversely, 1,4-products **5** were accessed with high selectivity and in moderate yield upon switching to the bidentate, nitrogen-based ligand 4,4′-di-*tert*-butyl-2,2′-bipyridine (dtbpy) **L3**.

### 3.2. 1,2-Borofunctionalization processes

**Scheme 5:**
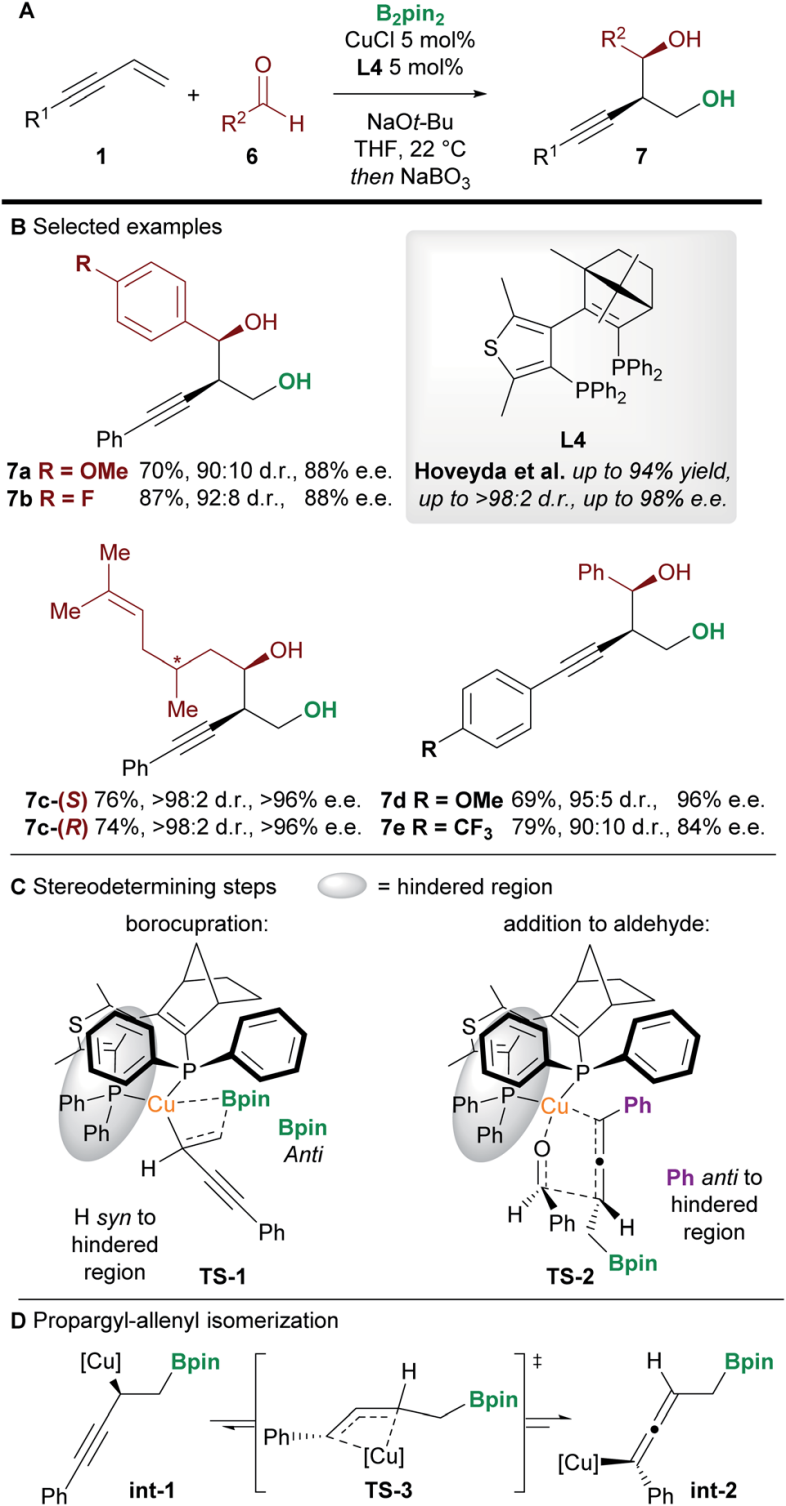
Hoveyda's 1,2-borofunctionalization of 1,3-enynes with aldehydes.

An important milestone in this field was achieved by Hoveyda and co-workers^16^ in 2014 when they realized the first enantioselective borylative 1,2-functionalization of 1,3-enynes **1** with aldehydes **6** (Scheme 5). The borylated products were oxidized *in situ* using NaBO_3_·4H_2_O to provide homopropargylic 1,3-diols **7** in good to excellent yield, and with high diastereo- and enantiocontrol. Aromatic and aliphatic aldehydes, and aryl-substituted enynes were all suitable coupling partners, but alkyl-substituted enynes proved more challenging substrates.

DFT studies were carried out on the two stereodetermining steps of this reaction (Scheme 5C). Firstly, the 1,2-borocupration was proposed as the enantiodetermining step of the process. This step proceeds through attack of a Cu-Bpin species at the C–C double bond of the enyne through a 4-membered transition state **TS-1**. Attack on the *Si* face of the enyne was favoured by 2.1 kcal mol^−1^ over attack on the *Re*-face. The resulting propargyl-copper species **int-1** can then isomerise *via***TS-3** to give the corresponding allenyl-copper species **int-2** (Scheme 5D). Although this step was not investigated in this report, the propargyl-allenyl isomerisation of transition metal complexes is a well-known phenomenon that has been studied using DFT calculations and X-ray crystallography.^17^ Finally, the desired product was proposed to form through the coupling of the allenyl copper species **int-2** and the aldehyde **6** in a diastereo determining, closed, 6-membered transition-state (**TS-2**, Scheme 5C). Other transition states (*e.g.* involving attack on the other face of the aldehyde) were modelled but were disfavoured by at least 1.9 kcal mol^−1^.

**Scheme 6:**
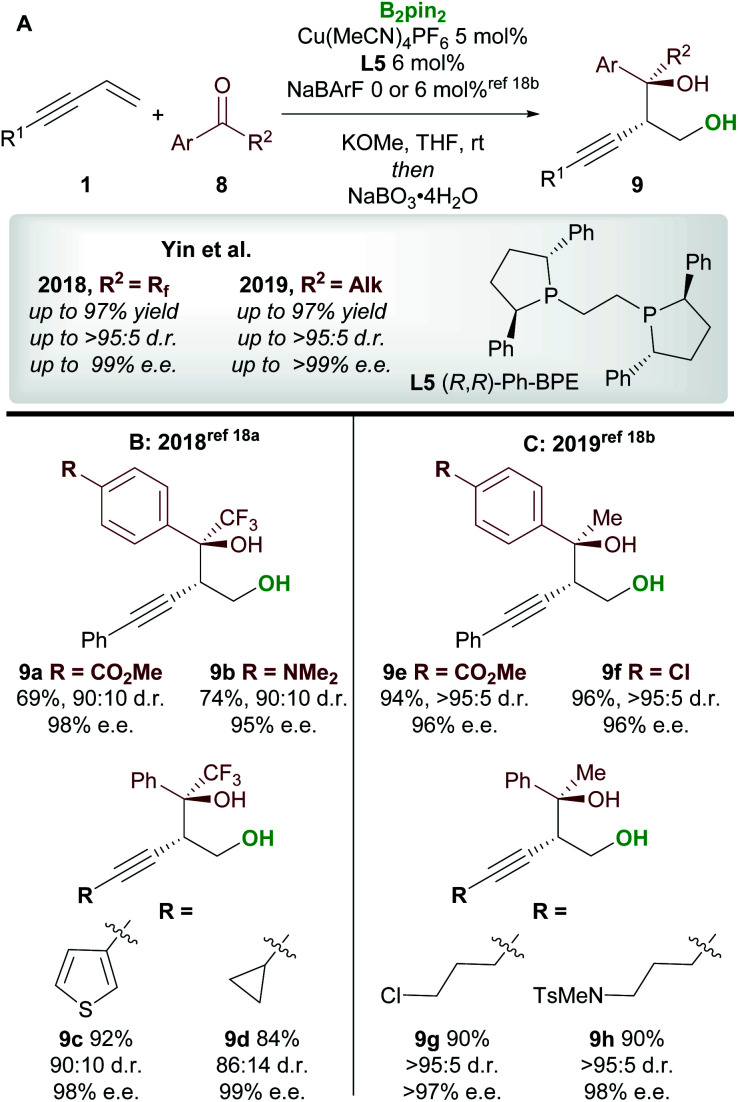
Yin's 1,2-borofunctionalization of 1,3-enynes with fluorinated ketones and aryl, alkyl-ketones.

Following on from this report, Yin and co-workers^18^ published consecutive papers on the copper-catalyzed borylative 1,2-functionalization of 1,3-enynes **1** with ketones **8** (Scheme 6A). Firstly, Jia, Yin *et al.*^18*a*^ described the coupling of aryl and alkyl-substituted enynes with perfluoroalkyl ketones (Scheme 6B). Using Ph-BPE **L5**, a ligand that has found much use in the field of copper-catalyzed functionalization of olefins, very good to excellent yields and excellent enantioselectivities were obtained across a wide range of aryl and alkenyl trifluoromethyl ketones. Similarly, longer perfluoroalkyl chains were also well tolerated. Yin and co-workers^18*b*^ then extended their work towards the use of aryl, alkyl ketones (Scheme 6C). The conditions were similar to those used in their previous report, however, the addition of a non-coordinating counter-anion NaBArF was required to obtain high yields. Excellent enantioselectivities were observed across all ketone inputs, and both aryl and alkyl-substituted 1,3-enynes were good substrates. In both reports, the authors chose to oxidize the products upon workup to afford the *anti*-homopropargylic alcohols **9**.

**Scheme 7:**
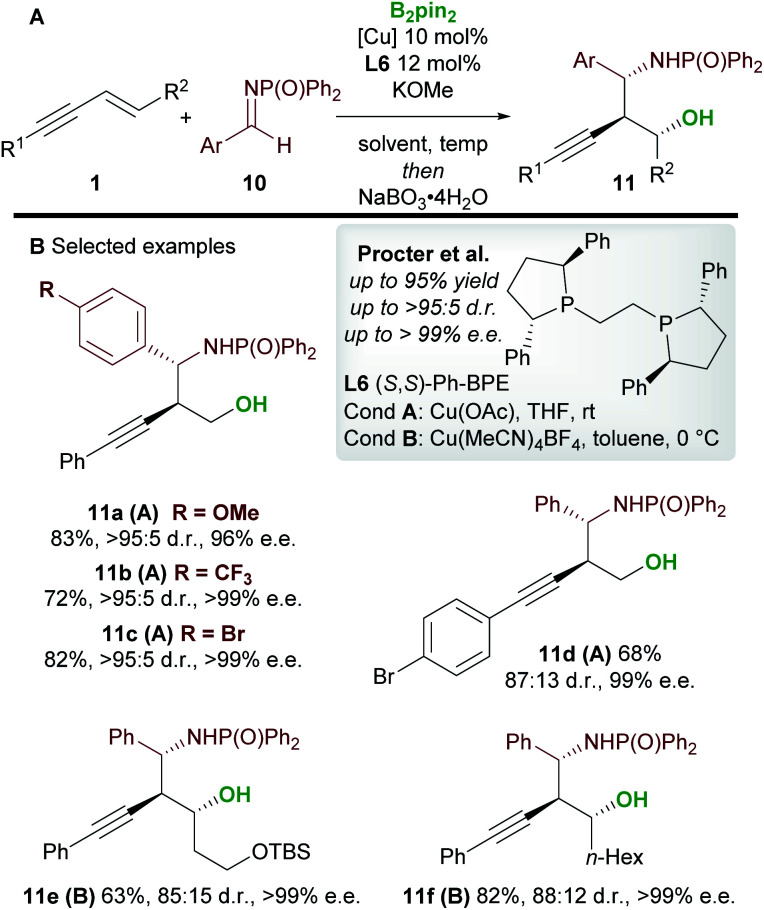
Procter's 1,2-borofunctionalization of 1,3-enynes with imines.

In 2020, Procter and co-workers^19^ developed the copper-catalyzed borylative 1,2-functionalization of 1,3-enynes **1** with aldimines **10** to give *anti*-homopropargylic amines **11** (Scheme 7). Interestingly, Ph-BPE **L6** was again the ligand of choice. A range of electron-rich and electron-deficient *N*-phosphinoyl imines coupled to enynes in very good yield and with excellent diastereo- and enantiocontrol. The authors found that a switch in solvent, temperature and copper catalyst ensured favourable reactivity with a wide range of enynes. The products were oxidized during work-up to afford the 1,3-aminoalcohols **11**. Importantly, the reaction was extended to 1,2-disubstituted (*E*)-enynes (**11e** and **11f**) to give products containing 3 contiguous stereocenters with high enantiocontrol.

### 3.3. 1,4-Borofunctionalization processes

**Scheme 8:**
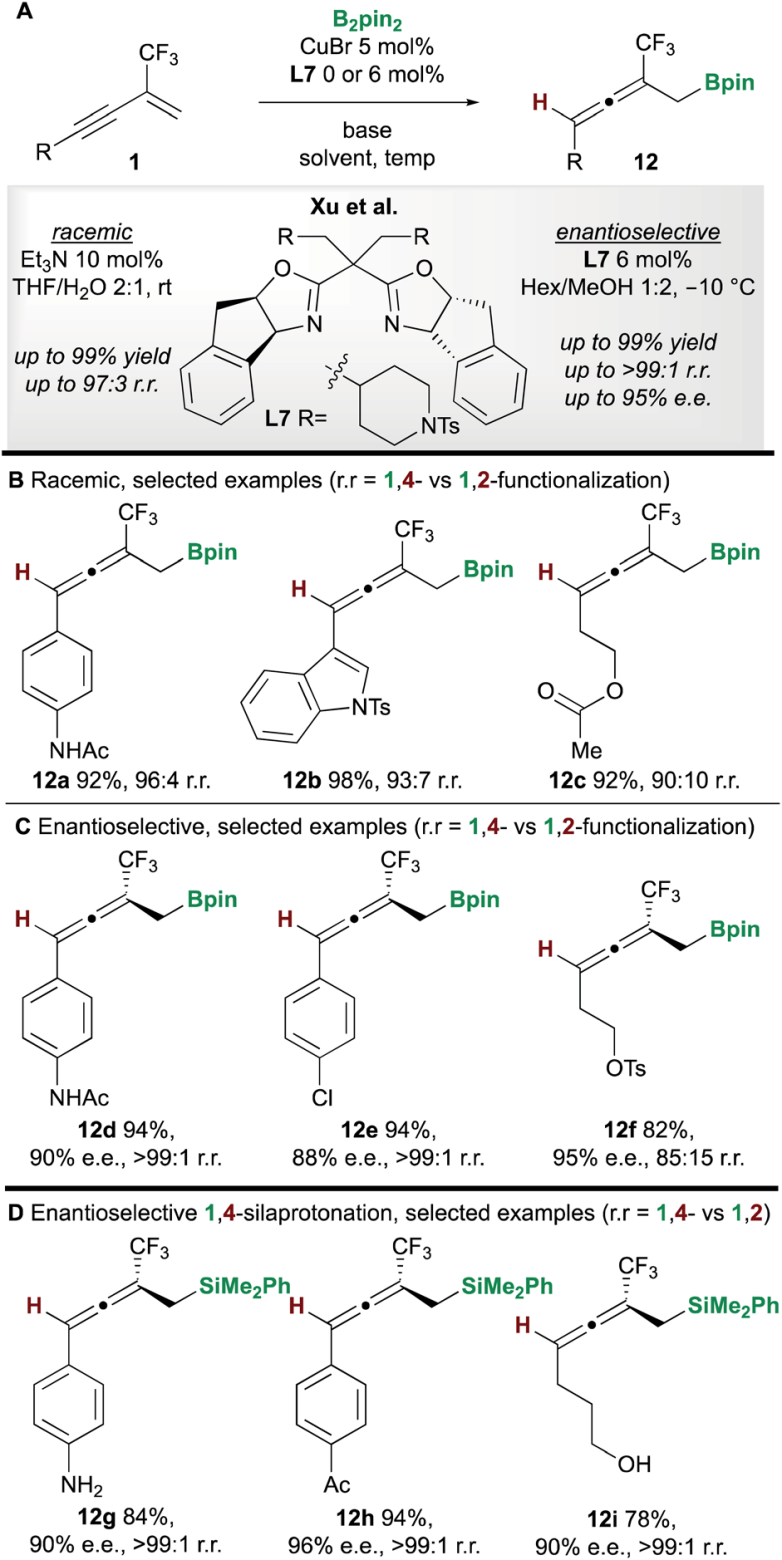
Xu's boroprotonation of 2-trifluoromethyl-1,3-enynes.

In 2020, Xu and co-workers^20^ developed the copper-catalyzed 1,4-boroprotonation of 2-trifluoromethyl-1,3-enynes **1**. A ligand-free CuBr catalyst promoted the formation of aryl and alkyl-substituted allenes **12** in very good to excellent yields (Scheme 8A and B). An efficient enantioselective process that, unusually, did not require a base was also developed using the bisoxazoline ligand **L7** (Scheme 8C). Interestingly, in some cases, the authors observed minor amounts of the 1,2-boroprotonated product, although no clear explanation for variation in regioselectivity was put forward. Furthermore, a related 1,4-silaprotonation operating under similar conditions exhibited high functional group tolerance and allowed access to the 1,4-silaprotonated products **12g–12i** with high enantiocontrol (Scheme 8D).

**Scheme 9:**
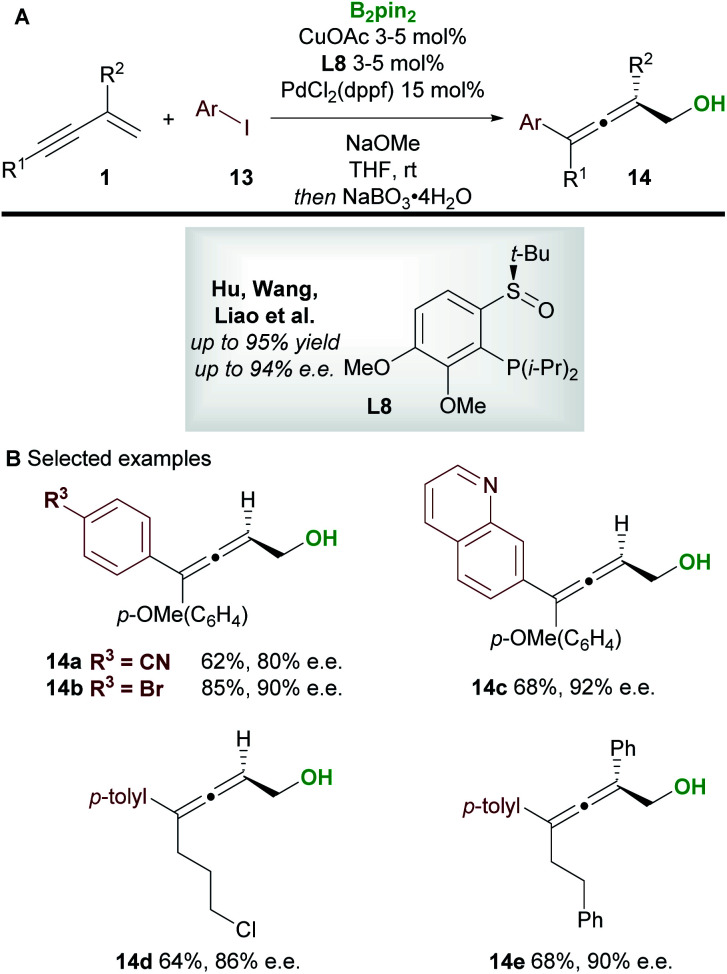
Hu, Wang, Liao's boroarylation of 1,3-enynes with aryl iodides.

In 2020, Hu, Wang, Liao, and co-workers^21^ disclosed a borylative copper palladium co-catalyzed 1,4-boroarylation of 1,3-enynes **1** (Scheme 9). Using their previously developed sulfoxide–phosphine (SOP) ligand **L8**, in conjunction with CuOAc (3 to 5 mol%), PdCl_2_(dppf) (15 mol%) and aryl iodides **13**, they obtained tri- and tetra-substituted enantioenriched allenes **14**. The coupling of electron-rich/neutral, aryl substituted 1,3-enynes (R^1^ = aromatic, R^2^ = H) with a wide range of aryl iodides gave tri-substituted allenes (**14a–14c**) in high yield and with high enantiocontrol. When using 1,3-alkylenynes (R^1^ = Alk), both tri-substituted (R^2^ = H, **14d**), and tetra-substituted (R^2^ = Ar, **14e**) products were accessible with very good enantiocontrol. It was proposed that the copper catalyst and chiral SOP ligand promoted an enantioselective 1,2-borocupration and subsequent isomerisation gave an allenyl copper species (*c.f.***int-2**, Scheme 5D). Then, after transmetallation, palladium catalyzed arylation gave the desired allene products **14**.

### 3.4. 3,4-Borofunctionalization processes

**Scheme 10:**
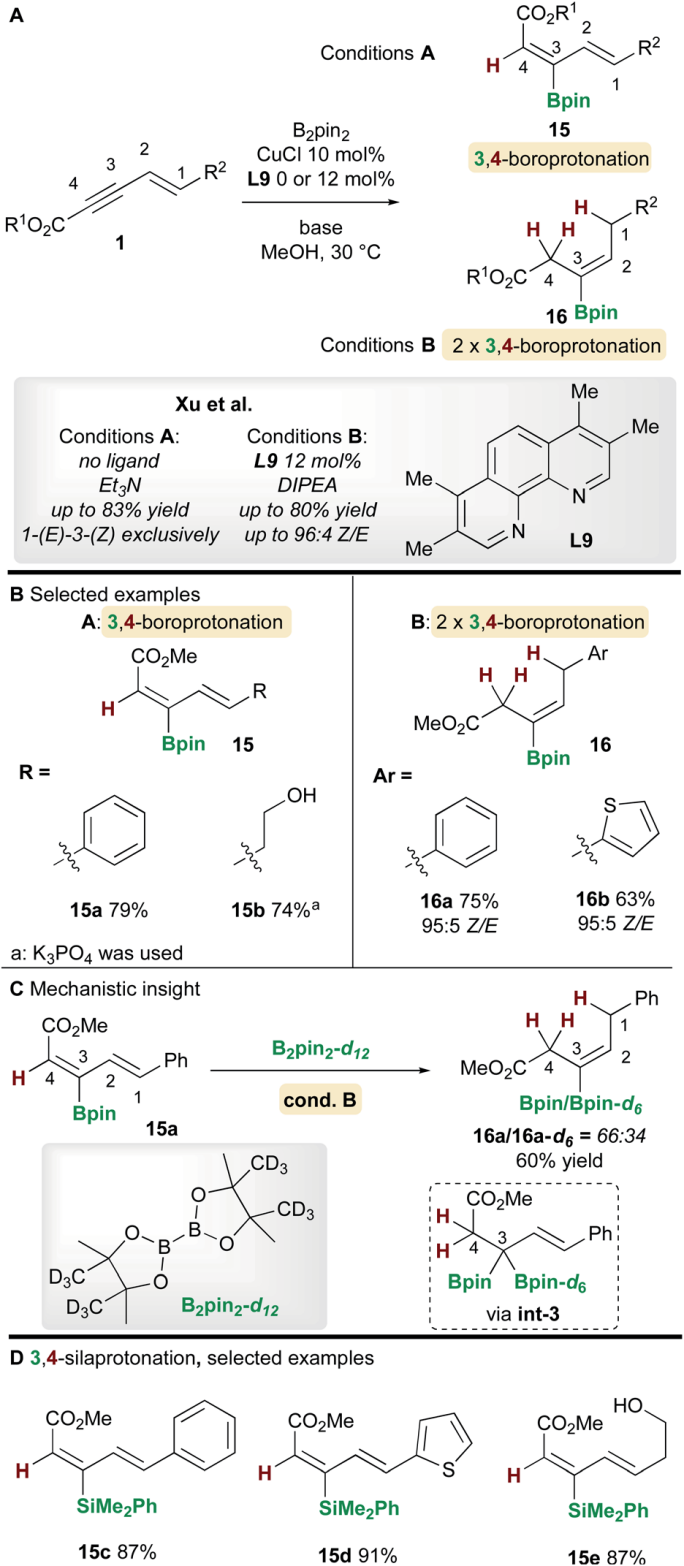
Xu's boroprotonation of 1,3-enynoates.

In 2019, Xu and co-workers^22^ described a copper-catalyzed boroprotonation of 1,3-enynoates **1** (Scheme 10). When a ligand-free CuCl catalyst and a substoichiometric amount of base were used in MeOH, the diene **15** from 3,4-boroprotonation was isolated with essentially perfect *E*,*Z*-selectivity (Scheme 10, cond. A). Moderate to very good yields were obtained with aryl- and alkyl-substituted 1,3-enynoates (Scheme 10B). Conversely, when using CuCl and 3,4,7,8-tetramethyl-1,10-phenanthroline ligand **L9** in the presence of 2 equivalents of di-isopropylethylamine (DIPEA), alkene products **16** were formed. Very good yields and *Z*-selectivities were obtained in this process when using (hetero)aryl-substituted 1,3-enynoates. It was proposed that product **16** forms through two rounds of boroprotonation across the C3/C4 bond of the enyne, followed by protodeborylation *i.e.* the 3,4-boroprotonated product **15** is formed initially, but undergoes another boroprotonation followed by protodeborylation. A control experiment lent support to this hypothesis: when 3,4-boroprotonated product **15a** was subjected to conditions B in the presence of B_2_pin_2_-*d*_12_, product **16a** was obtained in 60% yield with significant Bpin-*d*_6_ incorporation at the 3 position. This indicated that boroprotonation occurs across the C3/C4 bond of **15a** to give a 3,3-diborylated intermediate **int-3**, followed by an unselective protodeborylation to give a mixture of Bpin/Bpin-*d*_6_ products. A 3,4-silaprotonation using the ligand-free system was also developed (Scheme 10D). Products **15c–e**, were isolated with complete *E*,*E*-selectivity.

## 4. Hydrofunctionalization

With origins in the 1850s,^23^ copper hydride chemistry gained popularity with the introduction of Stryker's reagent^24^ [(PPh_3_)CuH]_6_ and its use for the selective reduction of carbonyl derivatives. Significant advancements by Buchwald,^25^ Lipshutz^26^ and others^23^ led to catalytic asymmetric reactions using copper hydride species. The application of copper hydride chemistry in processes involving enynes is particularly appealing as it allows facile access to propargyl and allenyl metal species that react selectively under mild conditions.

### 4.1. 1,2-Hydrofunctionalization processes

**Scheme 11:**
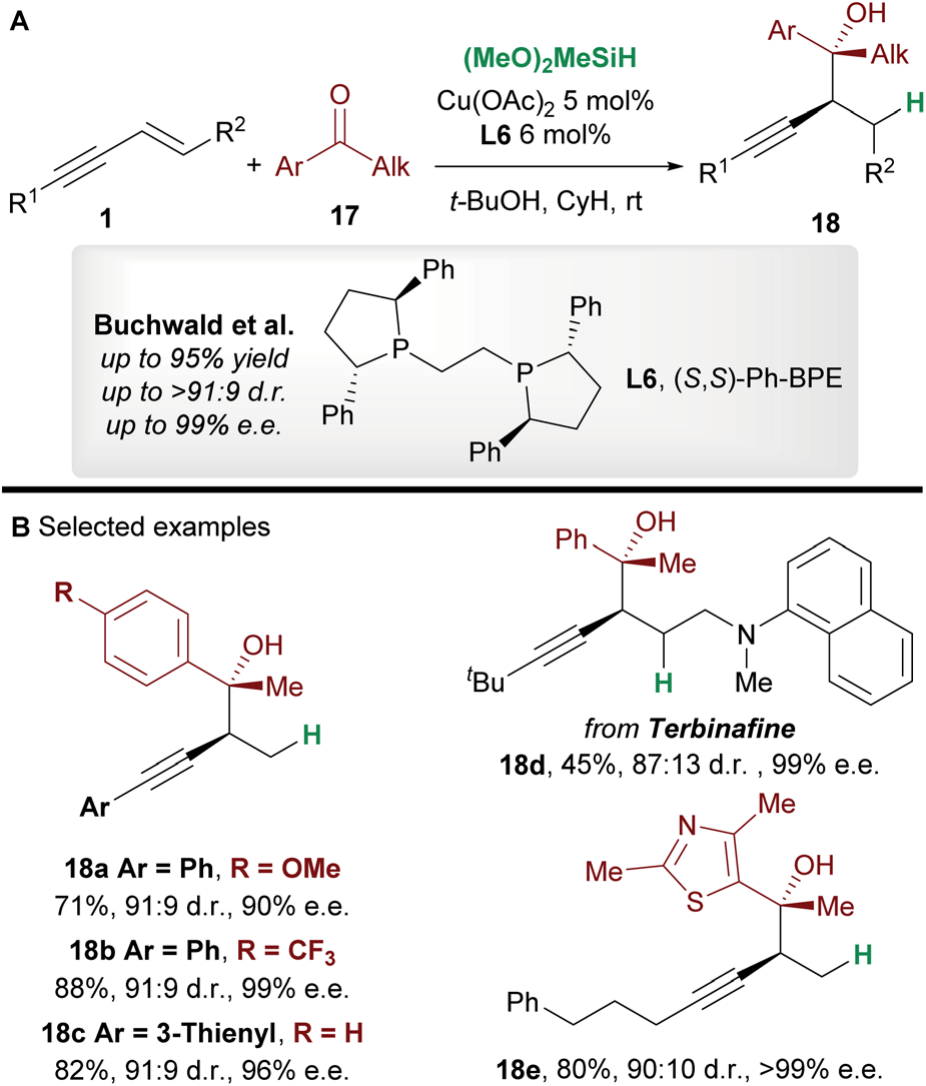
Buchwald's hydrofunctionalization of 1,3-enynes with ketones.

In 2016, Buchwald and co-workers^27^ reported an efficient copper-catalyzed 1,2-hydrofunctionalization of enynes **1** with ketones **17** to give highly substituted and enantioenriched homopropargyl alcohols **18** (Scheme 11A). The suitability of various enyne inputs was demonstrated and the process showed excellent diastereo- and enantioselectivity. The reaction tolerated both electron-donating and electron-withdrawing substituents on the aryl ring of the ketone. Notably, the antifungal medication terbinafine, which contains an enyne unit, responded well to hydrofunctionalization to give **18d** (Scheme 11B).

**Scheme 12:**
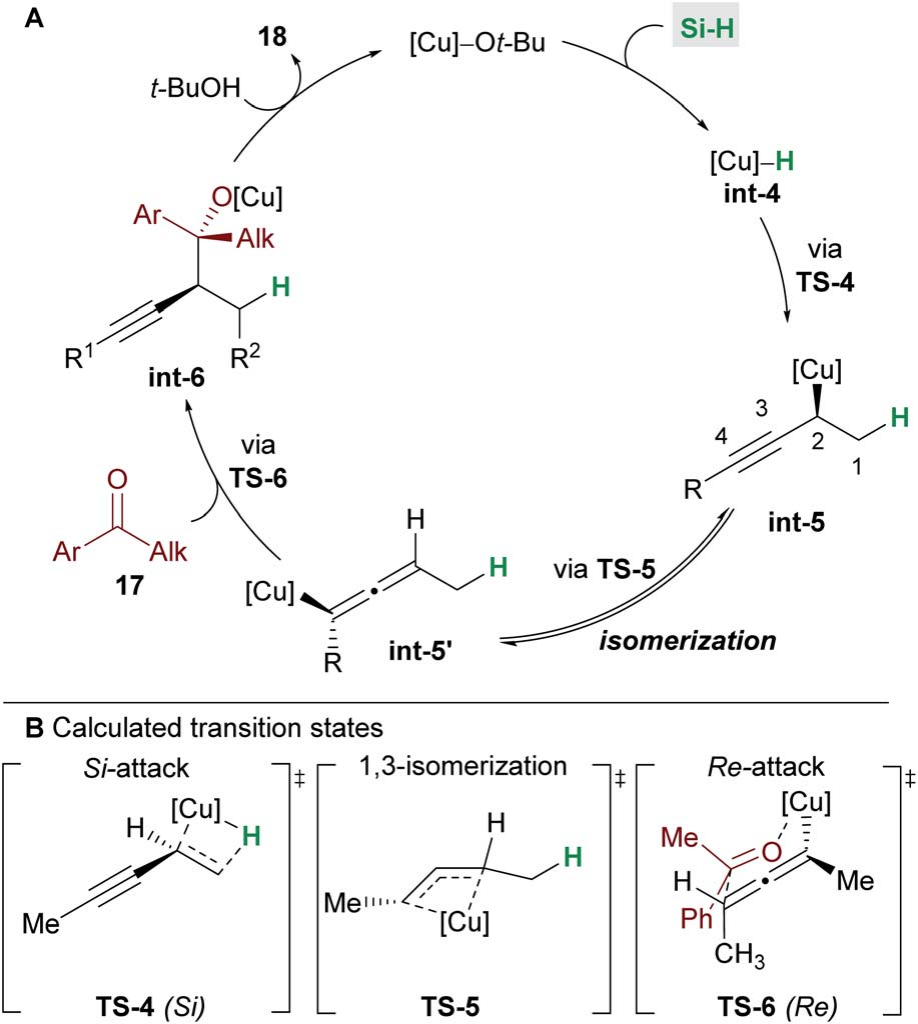
Buchwald's catalytic cycle (A) and DFT calculations (B).

A full mechanistic profile for the reaction was provided by DFT calculations (Scheme 12). The catalytic cycle begins with an enantioselective 1,2-hydrocupration of the enyne by the *in situ* generated copper hydride **int-4**, to give propargyl-copper species **int-5***via* a 4-membered transition state **TS-4**. It was found that attack on the *Si* face of the olefin was favoured by 5.5 kcal mol^−1^ and that the selectivity arises from minimisation of steric interactions between the alkynyl group and the phenyl groups of ligand **L6**. A facile, exergonic, and stereospecific 1,3-isomerization of **int-5** through **TS-5** led to the thermodynamically more stable allenyl-copper intermediate **int-5′**. The intermediate **int-5′** then undergoes coupling with the *Re* face of the ketone **17** through a 6-membered cyclic transition state **TS-6** to afford **int-6**. This step was rendered highly diastereoselective through the minimisation of unfavourable gauche interactions between the ketone and **int-5′**, and between the ligand and the allenyl substituents. Protonation of **int-6** delivers the homopropargyl alcohol products **18**. Clear parallels can be drawn between this mechanism and that proposed by the Hoveyda group (Scheme 5). These mechanisms also likely underpin the transformations in Schemes 6 and 7.

**Scheme 13:**
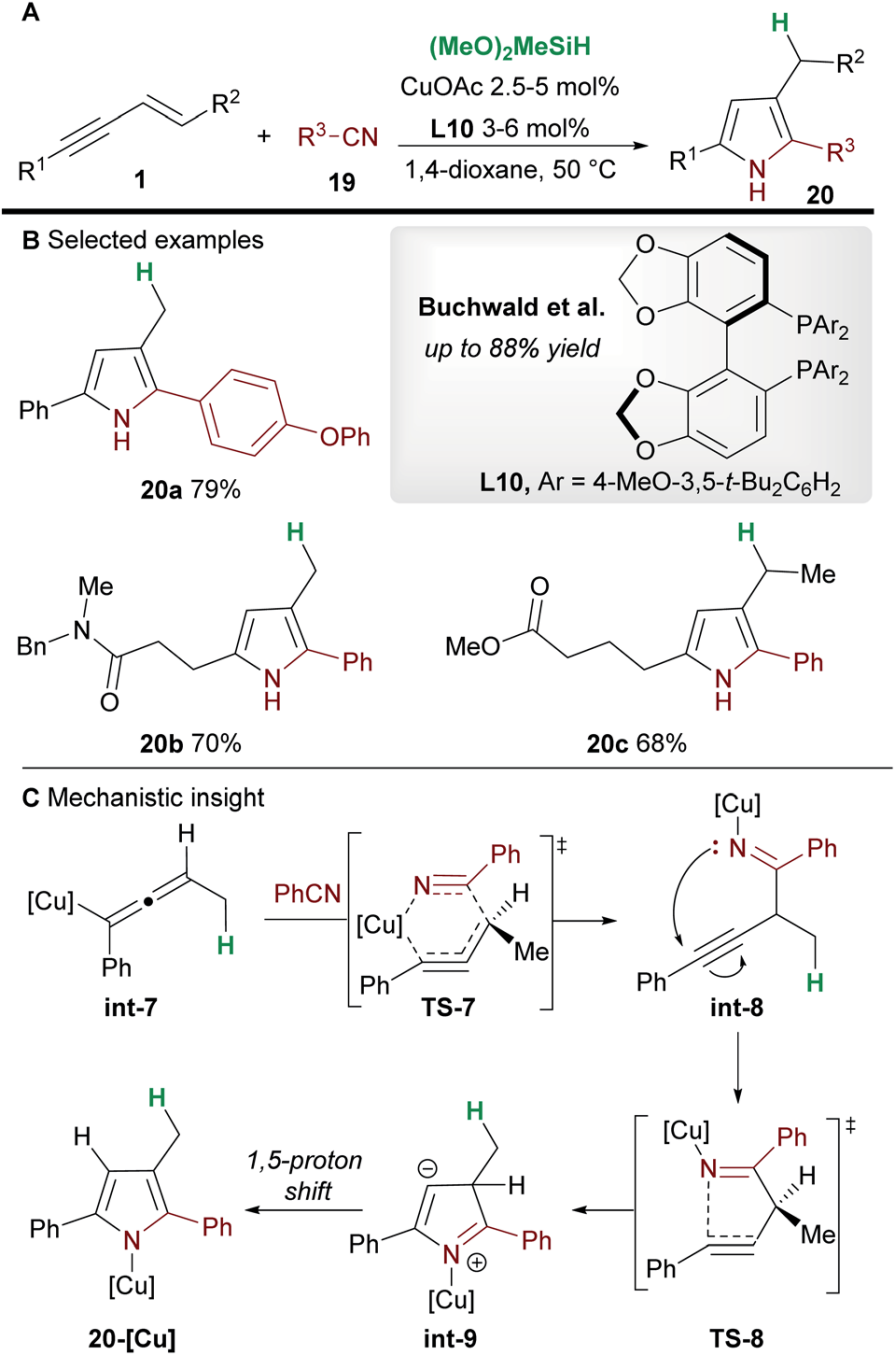
Buchwald's hydrofunctionalization of enynes with nitriles for the synthesis of pyrroles.

In 2020, Buchwald and co-workers^28^ used the copper-catalyzed 1,2-hydrofunctionalization of 1,3-enynes **1** with nitriles **19** to afford polysubstituted pyrroles **20** (Scheme 13). Both alkyl and aryl-substituted enynes, and aromatic and aliphatic nitriles afforded 2,3,5-trisubstituted pyrroles in moderate to good yield.

The mechanism of the reaction was investigated by DFT (Scheme 13C). As previously described (Scheme 12), hydrocupration and 1,3-isomerization to the allenyl copper **int-7** was found to be facile and exergonic. Subsequent coupling with nitrile **19** proceeds *via* the 6-membered transition state **TS-7** to deliver the 1,2-functionalized species **int-8** and is reminiscent of the addition to imines (Scheme 7). In related procedures, the catalytic cycle would usually close at this point to give functionalized (homo)propargylic products, however, in this case, a cyclization through transition state **TS-8**, followed by a 1,5-proton shift gave substituted pyrroles **20**. Small quantities of a side product resulting from 1,4-functionalization were also observed during the reaction.

### 4.2. 1,4-Hydrofunctionalization processes

**Scheme 14:**
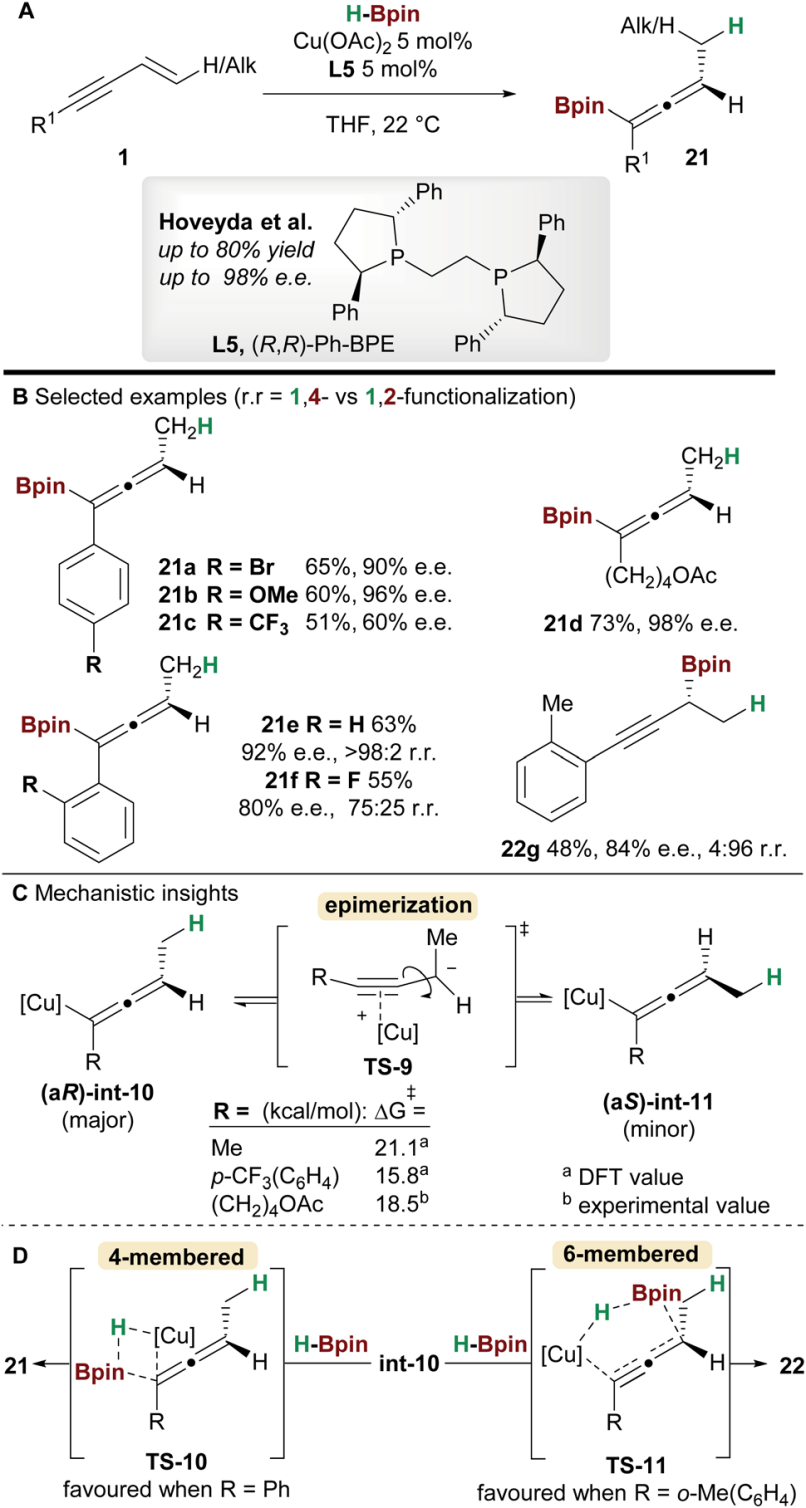
Hoveyda's hydrofunctionalization of 1,3-enynes with pinacolborane.

In 2018, an enantioselective copper-catalyzed hydroboration of 1,3-enynes **1** was developed by Hoveyda and co-workers^29^ using pinacolborane (HBpin) to synthesise substituted allenyl-Bpin products **21** (Scheme 14). Once again Ph-BPE **L5** was the ligand of choice for this reaction. Electron-rich and electron-neutral aryl/alkyl-substituted 1,3-enynes delivered allene products with excellent enantiocontrol, however, the process was less efficient for electron-poor substrates such as **21c**. Furthermore, 1,3-enynes bearing a bulky *ortho*-substituent on the aromatic ring afforded propargylic products, suggesting that steric hindrance about the alkynyl bond plays an important role in selectivity (products **21e**, **21f**, and **22g**).

**Scheme 15:**
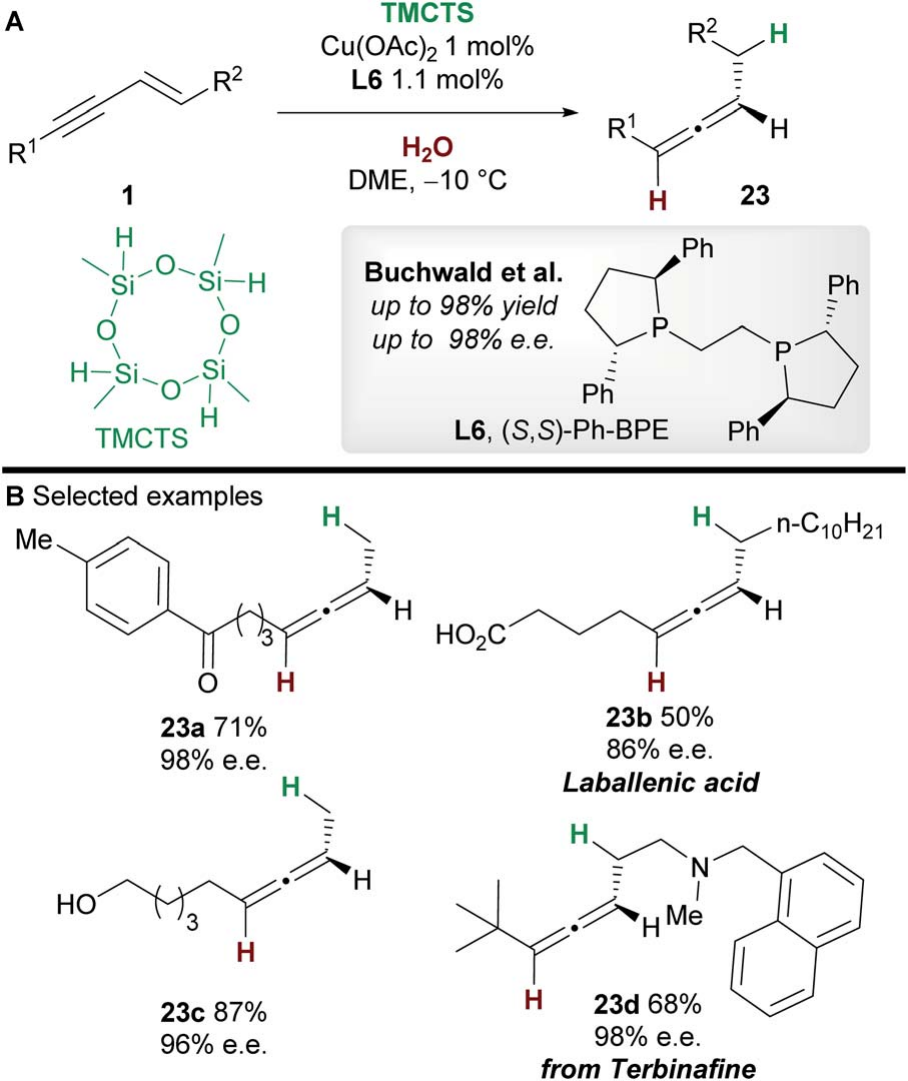
Buchwald's hydrofunctionalization of 1,3-enynes with TMCTS and water. TMCTS = 2,4,6,8-tetramethylcyclotetrasiloxane.

Intrigued by the lower enantioselectivities observed for electron poor substrates, mechanistic experiments and DFT calculations were conducted and the results provided important insight into the stereochemical integrity of the allenyl-copper intermediate (Scheme 14C). In agreement with the work of Buchwald *et al.* (Scheme 12), hydrocupration of the (*Re*)-face of the enyne was found to be enantiodetermining. A facile, stereospecific 1,3-isomerization then led to the allenyl-copper species **int-10**. The lower enantioselectivities observed for some substrates was proposed to arise from epimerization of intermediate **int-10***via* the propargyl anion-type transition state **TS-9**. Furthermore, this epimerization was shown to be more facile for electron-poor enynes *e.g.* that give **21c** (Scheme 14C). Low-temperature NMR kinetic studies on the epimerization of the related allenyl-copper species derived from the substrate that gives **21d**, found the epimerization barrier to be similar to those calculated by DFT (Scheme 14C). Finally, formation of the allenyl-Bpin product **21** and regeneration of the copper-hydride catalyst occurs *via* the 4-membered transition state **TS-10**. Conversely, when sterically demanding substituents are present, the corresponding 6-membered transition state **TS-11** was favoured and delivered the propargylic product *e.g.***22g** (Scheme 14D).

In 2019, an asymmetric semireduction of 1,3-enynes **1** was described by Buchwald and co-workers^30^ using 2,4,6,8-tetramethylcyclotetrasiloxane (TMCTS) as a hydride source. As is now established in these processes, the Ph-BPE ligand **L6** provided the best results. A range of aryl and alkyl-substituted 1,3-enynes, including those bearing sensitive functional groups, gave the allene products **23** with high enantioselectivity. Furthermore, the methodology was applied in an enantioselective synthesis of the natural product laballenic acid **23b**, without the need for protection of the acid group.

**Scheme 16:**
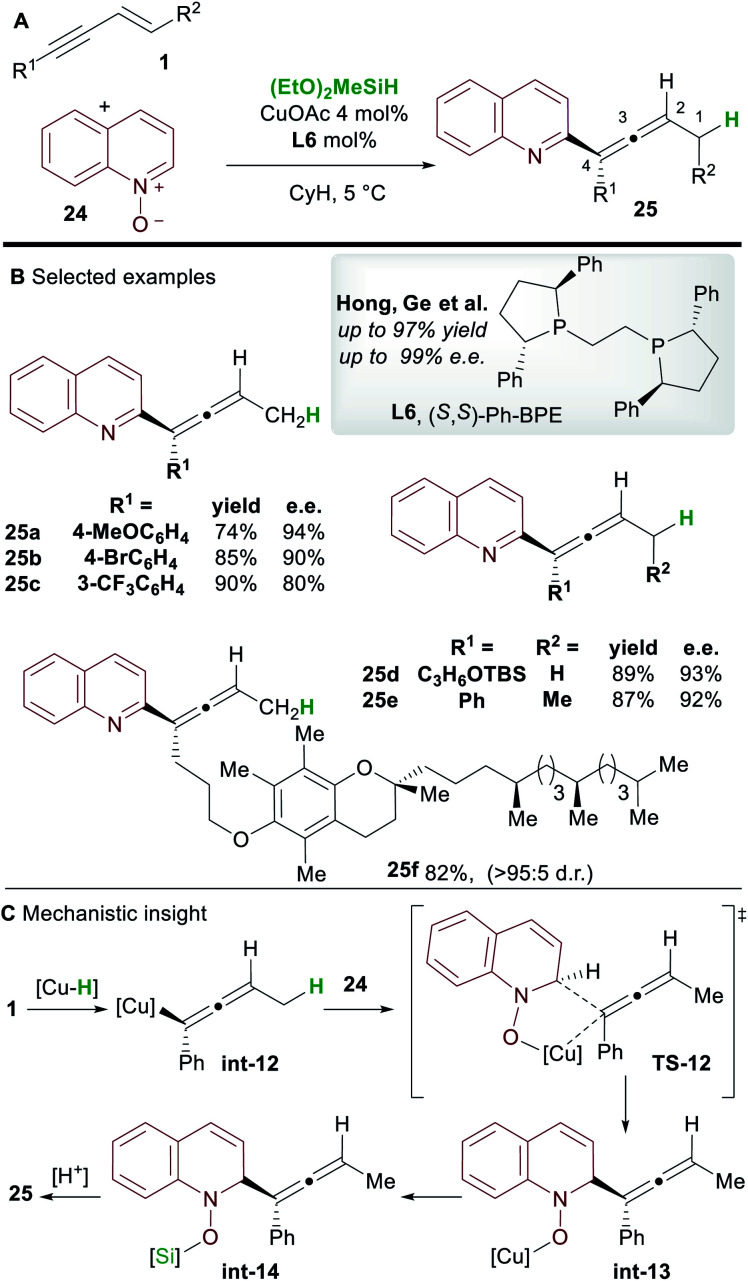
Hong and Ge's hydrofunctionalization of 1,3-enynes with quinoline *N*-oxide.

In 2018, Hong, Ge and co-workers^31^ reported the copper-hydride catalyzed allenylation of quinoline *N*-oxide **24** using enynes **1** and affording enantioenriched *N*-heteroaryl-substituted allenes **25** (Scheme 16). In this case also, the Ph-BPE ligand **L6** provided the best results. Aryl and alkyl-substituted 1,3-enyne inputs, as well as various heterocyclic *N*-oxides, gave heteroaryl substituted allene derivatives with high enantioselectivity. Aryl-substituted 1,3-enynes bearing electron-donating (**25a**) and electron-withdrawing groups (**25c**) were tolerated, although, as seen by Hoveyda and co-workers (Scheme 5), electron-poor and *ortho*-substituted aryl-1,3-enynes gave lower enantioselectivities (**25c**). Compatibility with some natural products, such as a vitamin E (to give **25f**) and a cholesterol derivative, was also shown. DFT calculations showed that, in accordance with previous reports by Buchwald and Hoveyda, stereospecific isomerization follows enantioselective 1,2-hydrocupration to give the allenyl copper complex **int-12**. Coupling of allenyl-copper **int-12** with the *N*-oxide was proposed to go through a 5 membered-transition state **TS-12** to give **int-13**. A related 7-membered transition state leading to the propargylic product was found to be disfavoured by 3.5 kcal mol^−1^. Finally, The N–O silylated adducts **int-14** re-aromatized spontaneously during work-up.

## 5. Copper-catalyzed radical functionalization of enynes

The addition of radicals to olefins is an essential tool for C–C bond formation in organic synthesis.^32^ Copper, with several readily accessible oxidation states, can mediate single- or two-electron processes and thus facilitate an array of redox processes that convert enynes to functionalized products.^33^

### 5.1. Regiodivergent processes

**Scheme 17:**
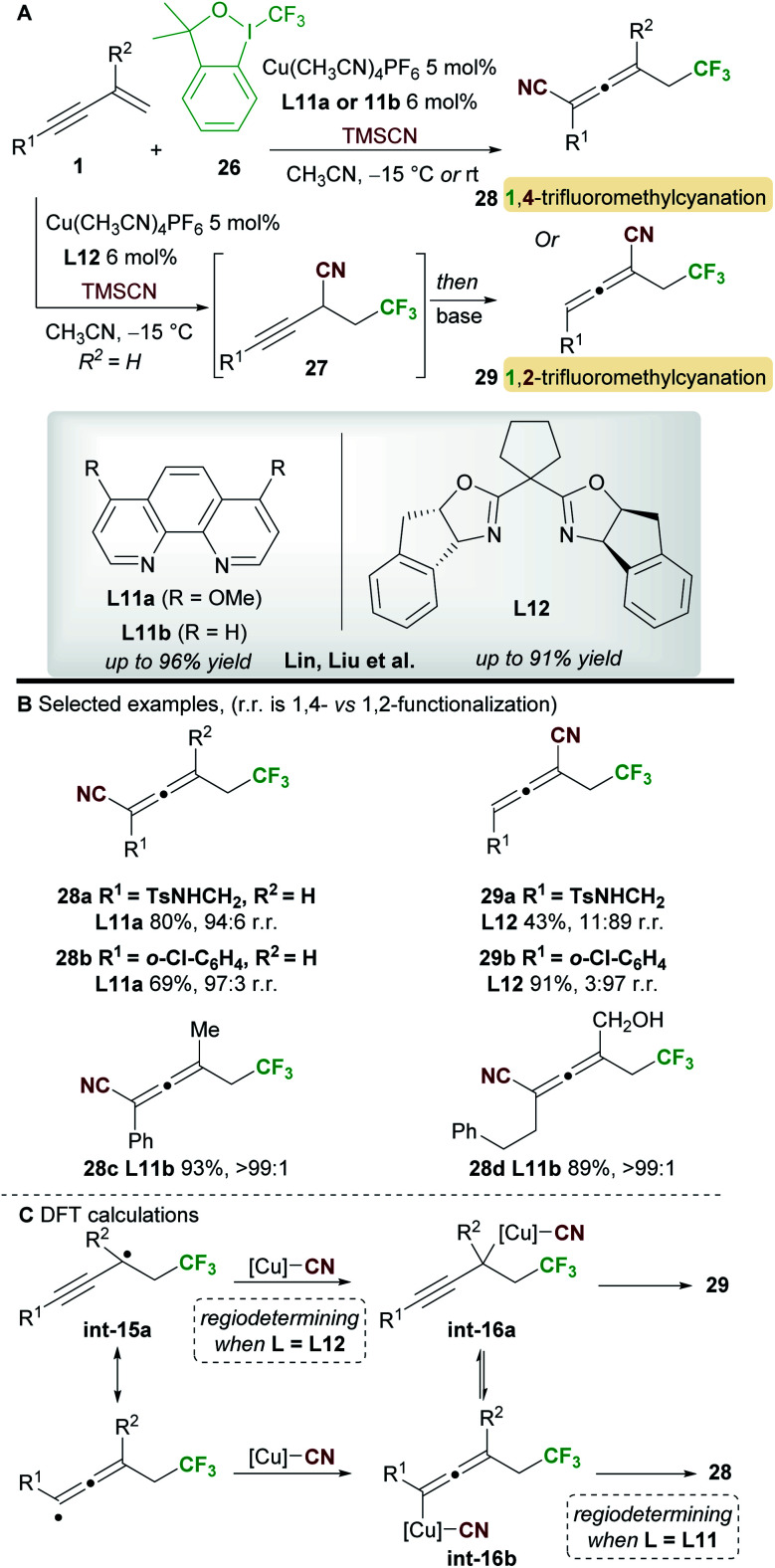
Lin and Liu's trifluorocyanation of 1,3-enynes.

In 2018, Lin, Liu and co-workers^34^ reported a ligand-controlled, regiodivergent trifluoromethylcyanation of 1,3-enynes **1** using Togni's reagent **26** and TMSCN (Scheme 17). Simple 1,3-enynes (R^2^ = H) were converted to either 1,4-trifluorocyanated allenes **28**, using a phenanthroline ligand **L11a**, or 1,2-trifluorocyanated allenes **29** using the bulkier bisoxazoline ligand **L12**. On the other hand, 2-substituted 1,3-enynes underwent 1,4-trifluoromethylcyanation only using ligand **L11b**, providing tetrasubstituted allenes **28**. The selectivity and yields remained high with substrates bearing various aromatic, heteroaromatic, and aliphatic alkynyl substituents (R^1^). The enantioselectivity of processes using the chiral ligand **L12** was not reported.

The authors studied the origin of regiodivergency using DFT (Scheme 17C). When using **L11**, delocalized radical **int-15a** can be trapped by the copper(ii)cyanide complex at both the propargyl and allenic positions. Thus, the regiodetermining step was found to be the reductive elimination, which proved easier for the allenyl-copper species **int-16b** leading to the observed 1,4-disubstituted allenes **28**. On the other hand, trapping of radical **int-15a** at the less congested, propargylic site with the bulky copper(ii) complex derived from **L12** was favoured. Thus, the authors suggest that regioselectivity is controlled by the reductive elimination step when using non-bulky ligands, while trapping of the radical **int-15a** determines the outcome of the reaction when using sterically demanding ligands.

### 5.2. 1,2-Functionalization

**Scheme 18:**
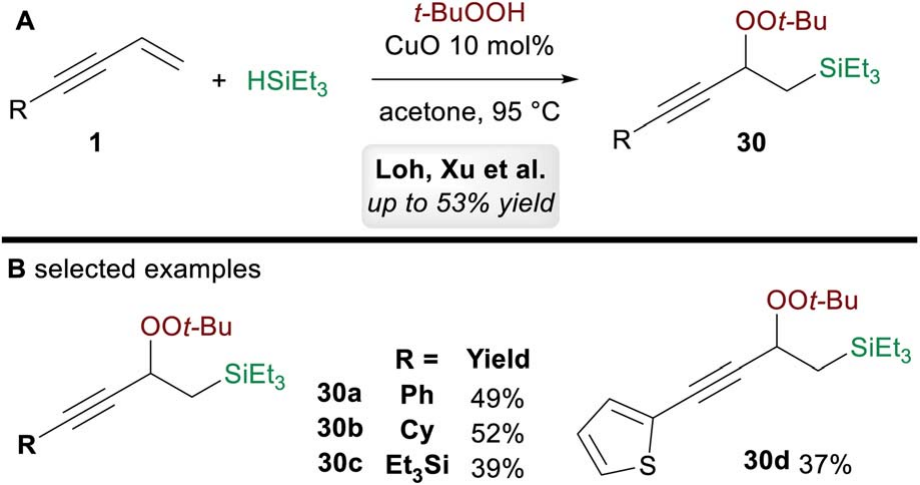
Loh and Xu's silylperoxidation of 1,3-enynes.

In 2017, Loh, Xu and co-workers^35^ disclosed a ligand-free copper-catalyzed regioselective 1,2-silylperoxidation of 1,3-enynes **1** using *tert*-butyl hydroperoxide (TBHP) and triethylsilane (Scheme 18A). Aryl, alkyl, and silyl-substituted 1,3-enynes were both compatible with the process and gave moderate yields of 1,2-functionalized products **30** (Scheme 18B). The process was also applied to other olefins such as α,β-unsaturated ketones, acrylates, and enamides.

### 5.3. 1,4-Functionalization

**Scheme 19:**
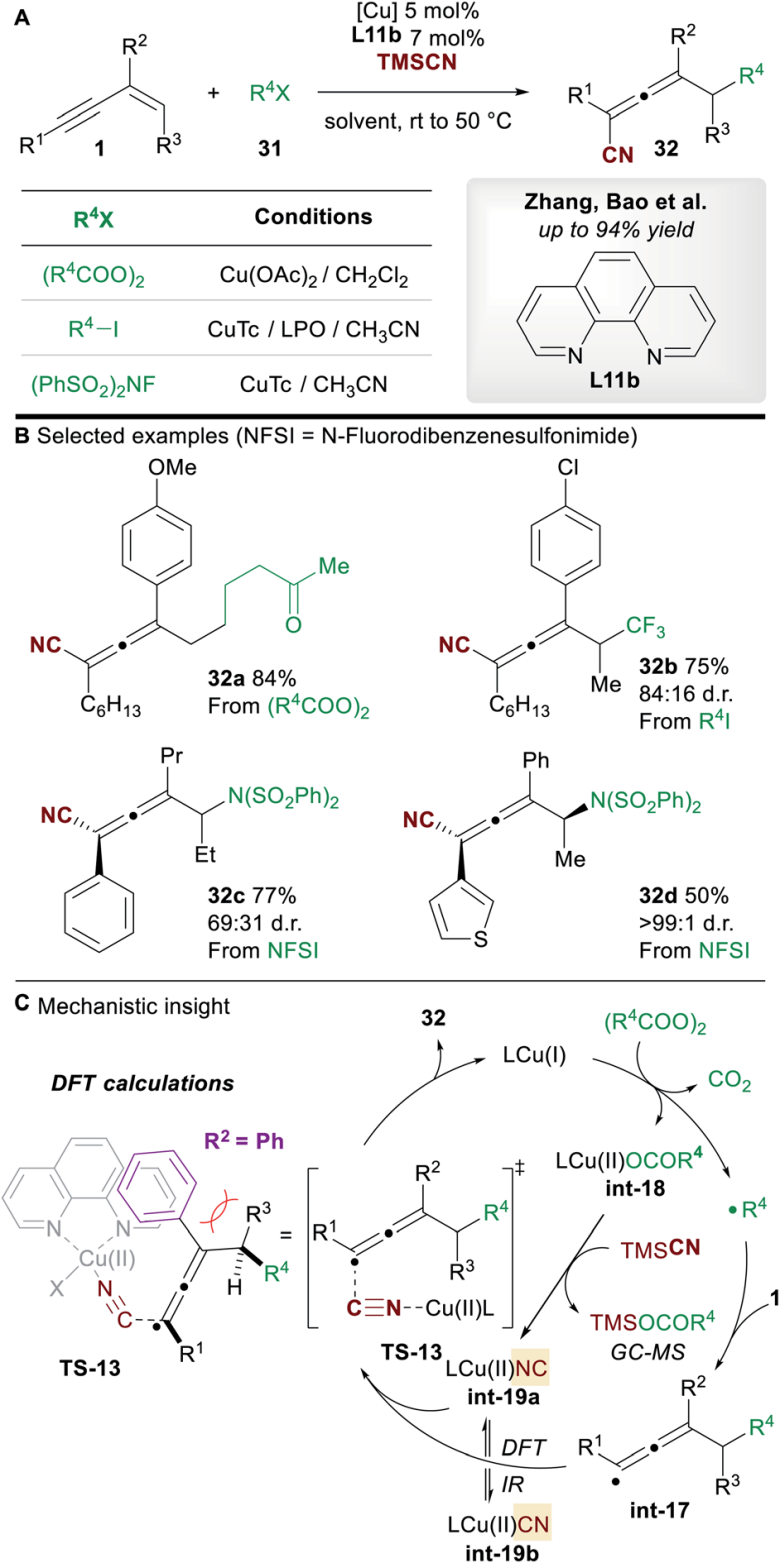
Zhang and Bao's carbocyanation of 1,3-enynes. NFSI = *N*-fluorobenzenesulfonimide.

In 2019, Zhang, Bao and co-workers^36^ reported a copper-catalyzed regioselective 1,4-carbo-/aminocyanation of 1,3-enynes **1** using trimethylsilyl cyanide (TMSCN) (Scheme 19A). A wide range of 1,4-carbocyanated allenes **32** was obtained using alkyl diacyl peroxide (to give **32a**) or alkyl iodide (to give **32b**) radical precursors **31** (Scheme 19B). 1,4-Aminocyanated allenes **32c** and **32d** were also obtained in good yields using *N*-fluorobenzenesulfonimide (NFSI) as the precursor of a N-centered radical. 2-Substituted enynes were used in all cases (R^2^ = aryl/alkyl). Substitution on the terminus of the alkene in the 1,3-enynes was also tolerated (R^3^ ≠ H). In these cases, diastereocontrol was found to be dependent on the nature of R^2^. The authors proposed a mechanism in which copper promotes the formation of a C-centered (or N-centered) radical (Scheme 19C), followed by radical addition to the alkene moiety of the 1,3-enyne to give allenyl radical **int-17**, whose intermediacy was ascertained by radical probe experiments. Interestingly, the cyanation was proposed to occur from a copper(ii)isocyano complex **int-19a** (detected by IR spectroscopy) rather than copper(ii)cyanide complex **int-19b**. This raises the possibility that an alternative mechanism for cyanation might be occurring in the report by Lin, Liu *et al.* (Scheme 17). DFT studies suggested that the energy barrier to cyanation through **TS-13**, from **int-19a**, was 13.5 kcal mol^−1^ lower in energy than the analogous step with **int-19b**. The higher diastereoselectivites observed when R^2^ = aryl were proposed to arise from a favourable π–π stacking interaction with the ligand 1,10-phenanthroline **L11b**.

**Scheme 20:**
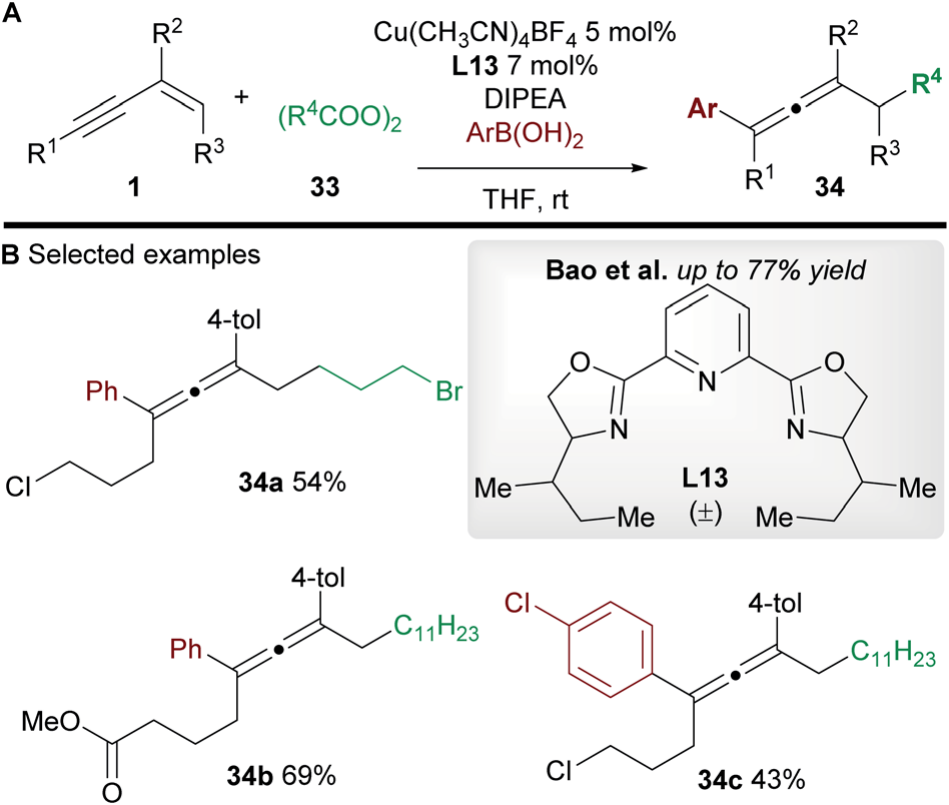
Bao's carboarylation of 1,3-enynes.

Bao and co-workers^37^ later reported a related transformation using aryl boronic acids in place of TMSCN to access 1,4-difunctionalized allenes **34** (Scheme 20A). A wide range of tetrasubstituted allenes bearing various aromatic and aliphatic groups was obtained in good yields (Scheme 20B). Mechanistic studies provided support for a radical mechanism and the authors suggested a catalytic cycle related to that shown in Scheme 19. Attempts to develop an asymmetric variant were unsuccessful.

## 6. Copper-catalyzed functionalization of 1,*n*-non-conjugated enynes

1,*n*-Non-conjugated enynes are important and versatile building blocks. For example, skipped enynes (1,4-enynes) have been used in the synthesis of important natural products,^38^ while other 1,*n*-enynes, following the work of Trost,^39^ have proved valuable in transition-metal catalyzed cycloisomerization reactions.^8*b*,8*c*^ Herein, we will survey the copper-catalyzed functionalization of 1,*n*-enynes.

**Scheme 21:**
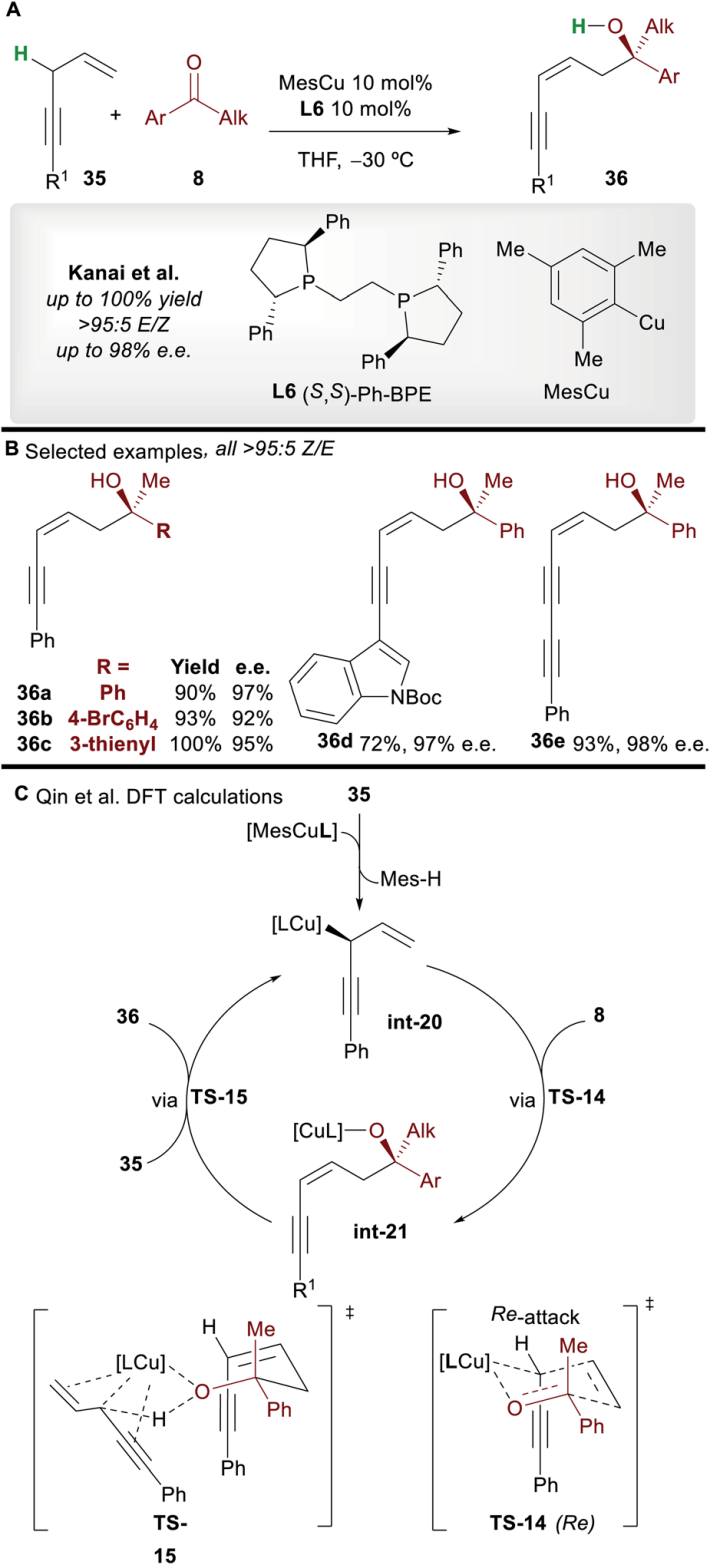
Kanai's functionalization of 1,4-enynes with ketones and Qin's subsequent DFT study.

The copper-catalyzed asymmetric addition of 1,4-enynes to ketones was reported by Kanai and co-workers^40^ in 2017 (Scheme 21A). Alkyl and aryl-substituted 1,4-enynes gave functionalized 1,3-enyne products **36** with complete *Z*-selectivity, good to excellent enantioselectivity, and in very good yield with the commonly used ligand **L6**. A wide range of functional groups was tolerated and no base was required; the 1,4-enynes are initially deprotonated by the **L6**·CuMes complex, and then by the copper-alkoxide species **int-21** formed during the catalytic cycle.

The mechanism was studied by Qin and co-workers^41^ using DFT (Scheme 21C). Calculations showed that pre-catalyst **L6**·CuMes deprotonates **35**, producing the allylcopper **int-20**. A 6-membered chair-like transition state **TS-14**, in which the larger phenyl group of the ketone occupies a pseudo-equatorial position and the *Re*-face of the ketone is attacked is invoked to account for the selectivity of the process. The cycle is closed by deprotonation of **35** by the copper-alkoxide complex **int-21***via***TS-15**. The deprotonation of the enyne *via***TS-15** was proposed to be the rate determining step of the process.

**Scheme 22:**
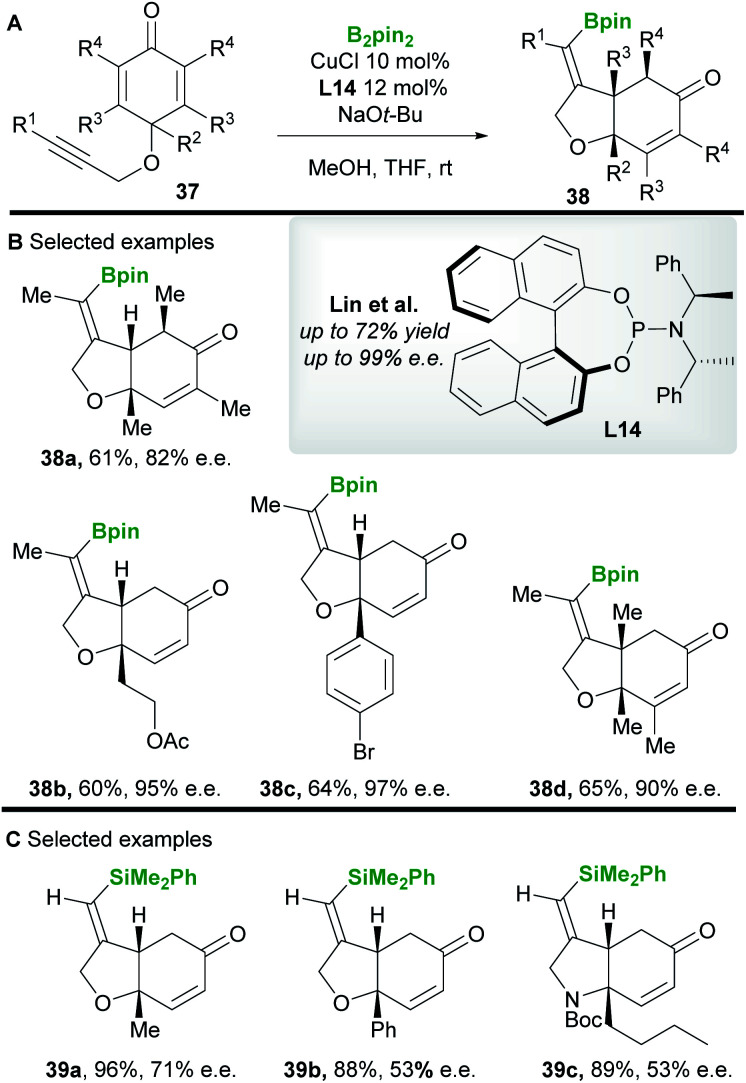
Lin's borylative/silylative cyclization of 1,6-enynes.

In 2013 Lin and co-workers^42^ reported a copper-catalyzed asymmetric borylative desymmetrization of cyclohexadienones **37** bearing a 1,6-enyne functional group. The cyclization reactions proceeded with high regio- and enantiocontrol to yield *cis*-dihydrobenzofuran derivatives **38** (Scheme 22A and B). *cis*-Products were exclusively obtained in moderate to good yield using the phosphoramidite ligand **L14**. Competing conjugate borylation is thought to be suppressed by the steric hindrance imposed by the neighbouring (R^2^) substituent. Furthermore, the regioselectivity of alkyne borylation is thought to arise from the oxygen in the tether, coordinating and directing copper to the β-position of the alkyne. In 2019, the same group^43^ (Scheme 22C) reported the silylative variant using Suginome's reagent (PhMe_2_Si-Bpin) and (*R*,*R*)-Ph-BPE **L5**.

**Scheme 23:**
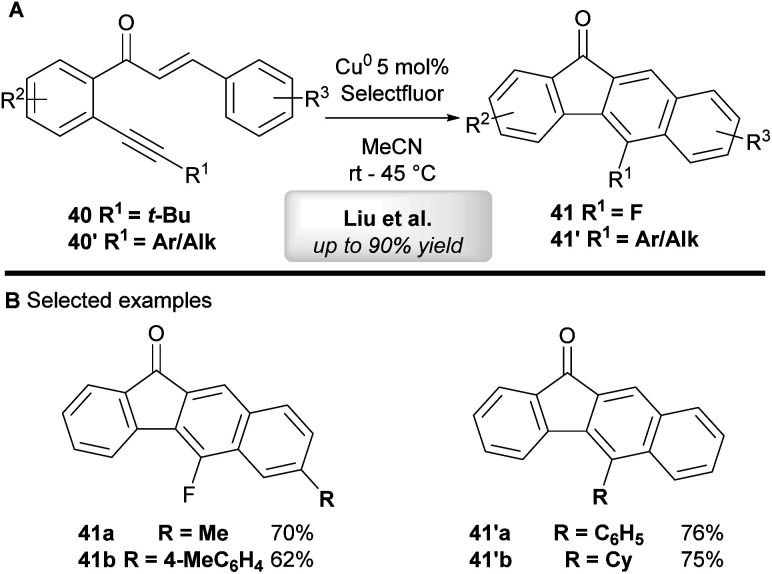
Liu's copper-catalyzed cyclization of chalcone derivatives.

Liu and co-workers^44^ have developed a copper-catalyzed tandem annulation of 1,6-enynes to give benzo[*b*]fluorenone derivatives (Scheme 23A). 5-Fluorinated benzo[*b*]fluorenones **41** were obtained when using *tert*-butyl-substituted alkynes **40** through a tandem annulation, C–(*t*-Bu) bond-cleavage and fluorination. On the other hand, aryl and other alkyl bearing 1,6-enynes **40′** exclusively provided 5-aryl-substituted benzo[*b*]fluorenones **41′** in moderate to excellent yield.

**Scheme 24:**
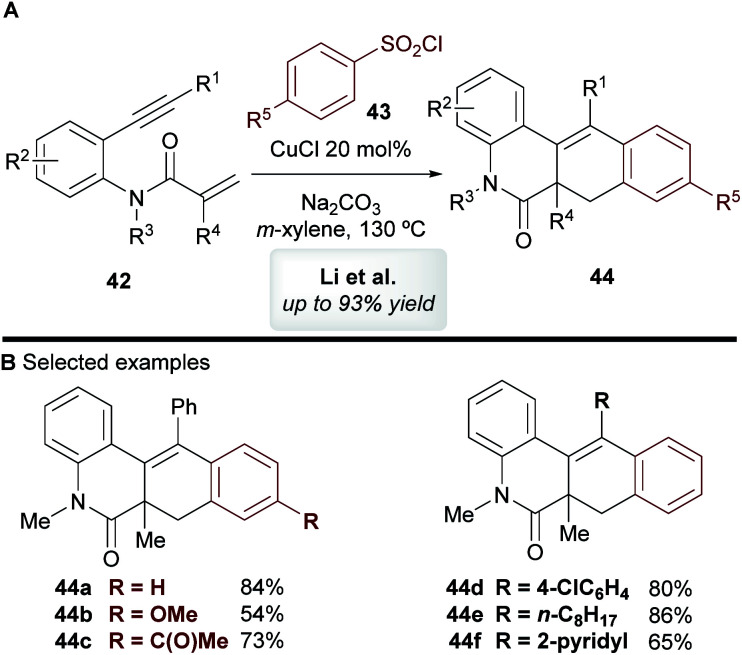
Li's cyclization of 1,7-enynes with sulfonyl chlorides.

In 2014, Li and co-workers^45^ disclosed the copper-catalyzed cascade cyclization of 1,7-enynes **42** using sulfonyl chlorides **43** as coupling partners, affording substituted benzo[*j*]phenanthridin-6(5*H*)-one scaffolds **44** (Scheme 24). The mechanism of this reaction was not explored by the authors, but they proposed that an aryl radical or aryl-copper species, formed from the aromatic sulfonyl chloride, first reacts at the alkynyl moiety, which triggers a cascade process to deliver products **44**

**Scheme 25:**
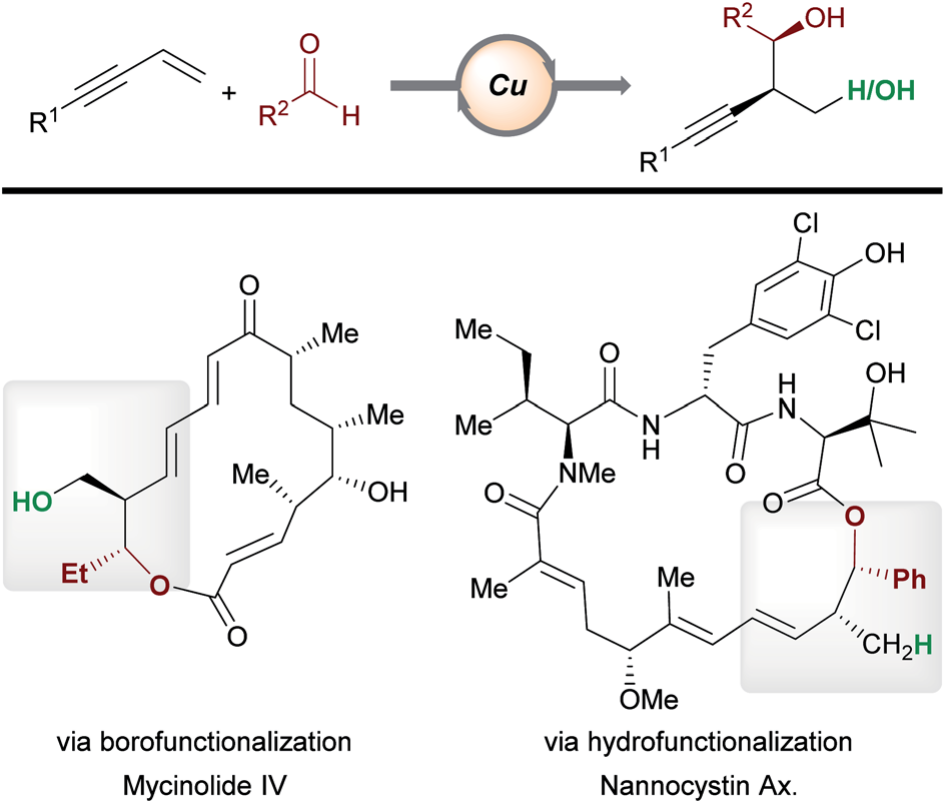
Applications in target molecule synthesis.

## 7. Conclusions

In recent years, copper-catalyzed transformations of readily available enynes have allowed the construction of densely functionalized scaffolds, often in an enantioselective fashion. Due to the ambident nature of enynes and the versatile reactivity of copper, a range of products are accessible by the careful marriage of substrate and catalyst class. We have highlighted examples of processes being used in the functionalization or preparation of bioactive molecules (*e.g.*Schemes 11, 15 and 16). In addition, copper-catalyzed functionalizations of enynes have begun to find application in total synthesis (Scheme 25). For example, Hoveyda *et al.*^16^ have constructed known fragments of tylonolide and mycinolide IV using the enantioselective borofunctionalization of enynes with aldehydes. Similarly, Fürstner, Müller and co-workers utilized a related hydrofunctionalization in an approach to the cytotoxic natural product nannocystin Ax.^46^ We expect to see further applications of copper-catalyzed enyne functionalization in future target molecule syntheses. To find widespread application, some key challenges in this area need to be overcome. For example, our understanding of, and our ability to predict, the regioselectivity of a given process will aid in synthesis planning. With regard to boro-/hydro-functionalization, methods that expand the scope of effective aromatic enyne substrates are needed, as electron-poor and hindered aromatic enynes are often unsatisfactory substrates (see Schemes 5, 14 and 16). Ultimately, the ability to design more efficient and more powerful processes will be key in a future shaped by sustainable catalysis.

## Conflicts of interest

There are no conflicts to declare.
